# Resistin—Can it be a new early marker for prognosis in patients who survive after a cardiac arrest? A pilot study

**DOI:** 10.1371/journal.pone.0210666

**Published:** 2019-01-16

**Authors:** Raluca Mihaela Tat, Adela Golea, Ştefan Cristian Vesa, Daniela Ionescu

**Affiliations:** 1 Department of Anesthesia and Intensive Care I, "Iuliu Haţieganu" University of Medicine and Pharmacy, Cluj-Napoca, Cluj, Romania; 2 Surgical Department of "Iuliu Haţieganu" University of Medicine and Pharmacy, Cluj-Napoca, Cluj, Romania; 3 Department of Pharmacology, Toxicology and Clinical Pharmacology, "Iuliu Haţieganu" University of Medicine and Pharmacy, Cluj-Napoca, Cluj, Romania; 4 Outcome Research Consortium, Cleveland, United States of America; International University of Health and Welfare, School of Medicine, JAPAN

## Abstract

**Aim:**

The aim of our study was to evaluate the potential role of resistin in estimating the 30 days prognosis in patients with hypoxic-ischemic organ injury who survived after a cardiac arrest (CA).

**Materials and methods:**

The study included 40 patients resuscitated after a non-traumatic out-of-hospital CA admitted in Emergency Department (ED). All patients were followed for 30 days after CA or until death. Clinical data on admission were recorded. Blood samples were collected on admission in ED (0-time interval), and at 6, 12, 24, 48- and 72-hours following resuscitation. Serum concentrations of resistin, S100B and neuron specific enolase (NSE) were measured. Several predictive scores for the mortality at 30 days were created with logistic regressions.

**Results:**

At each time interval, median serum levels of resistin and S100 B were significantly higher in non-survivors compared to survivors. For NSE, plasma levels were significantly lower in survivors as compared to non-survivors at 48 and 72 hours, respectively. Accurate predictive scores for 30-days mortality were the ones which included the values of resistin and S100B measured at 12 hours after admittance [AUC 0.938 (0.813–0.989), sensitivity 85.71% (67.3%– 96%), specificity 91.67% (61.5%’99.8%), p<0.001], which included the values of all three markers measured at 12 hours after admittance [AUC 0.955 (0.839–0.995), sensitivity 82.14% (63.1%’93.9%), specificity 100.00% (73.5%’100.0%), p<0.001] and the that included the values of resistin and S-100B at 6 hours together with serum lactate on admission [AUC = 0.994 (0.901–1.0), sensitivity 96.4% (81.7%’99.9%), specificity 100.00% (73.5%’100.0%), p<0.001].

**Conclusion:**

In our study, serum levels of resistin or a combination of resistin with S-100B or resistin with S-100B and lactate, were highly predictive for 30 days mortality in resuscitated patients after CA. Further studies on large number of patients are needed to confirm our data.

## Introduction

At present, hypoxic-ischemic organ injury after cardiac arrest (CA) remains a major health problem due to increased mortality, morbidity and length of hospital stay. Most of the hospital stay is pent in intensive care units, which leads to increased costs and implications for health system, patient and family. Majority of these patients have poor outcomes. Comatose patients with brain injury after out-of-hospital cardiac arrest (OHCA) have survival rate of approximately 30% [1–3].

Therefore, numerous attempts have been made to identify those patients with high chances of favorable outcome after return of spontaneous circulation (ROSC). In these cases efforts should be made to obtain full recovery. Thus, in the past years a number of studies focused on the potential role of biomarkers, along with clinical and imagistic criteria in estimating outcomes in patients who suffer an out-of-/in-hospital CA [4–8].

Neuron specific enolase (NSE), isomeric form of glycolytic enolase, is found almost exclusively in neurons and neuroendocrine cells. It is now accepted that increased levels of NSE in patients with hypoxic encephalopathy after CA are especially correlated with the severity of neurological injuries and less with the risk of death (the current recommendations of the guidelines are to make it a prognostic marker after the first 3 days of CA) [1–3, 9–11].

S100 protein family has an important regulatory role in different cellular processes. It has been shown that S-100B has a high sensitivity in detecting brain damages, but it can also be released from other injured sources such are adipocytes or myocardial cells [1, 9]. Increased plasma levels of S-100B protein were well correlated with the severity of post-anoxic neurological damages and with unfavorable outcome in patients with cerebral hypoxic injuries [2, 6, 9, 12, 13].

At the moment, it is not possible to correctly predict patients’ outcome after ROSC strictly based on these biomarkers due to their increased heterogeneity and variability in time [3, 8].

Resistin (ADSF- adipose tissue-specific secretory factor) is a protein belonging to resistin-like molecules family and is expressed in humans by a number of cells including adipocytes, peripheral blood mononuclear cells, macrophages and bone marrow cells [14, 15]. Recent studies have drawn attention on the potential role of resistin as a biomarker than can predict mortality in patients with cardio-vascular disease (in close connection with the ability of resistin to influence glucose and insulin metabolism, thrombosis, angiogenesis and smooth muscle cell dysfunction, but as a factor regulating expression of Vascular Cell Adhesion Molecule-1 on endothelial cells) and as a marker of inflammation that can predict survival in critically ill patients. At this point, this correlation was frequently associated with organ dysfunction in patients with sepsis [16–19], but its role in patients who have a successfully resuscitated CA (although in this case, inflammatory processes underlie hypoxic organs injury) is unknown.

The aim of our study was to evaluate the potential role of resistin in estimating 30 days prognosis in patients with hypoxic-ischemic organ injury who survived after a CA.

## Materials and methods

The study was prospective, analytical, longitudinal, observational and cohort type. It included all consecutive patients resuscitated after non-traumatic OHCA admitted to the Emergency Department (ED) of County Emergency University Hospital Cluj-Napoca, who were subsequently transferred in intensive care units, between May 2016 and October 2017. All patients were followed for 30 days after CA or until death. This study was approved by the Ethics Committee of "Iuliu Haţieganu" University of Medicine and Pharmacy, with registration number 59/14.03.2016. Informed consent for inclusion in the study was obtained from patients’ proxies in all cases. Inclusion criteria were age between 18–85 years and CA. Exclusion criteria were age under 18 and over 85 years, pregnancy, CA due to trauma, acute bleeding from non-traumatic condition, re-arrest with unsuccessful resuscitation within 6 hours from hospital arrival, arrest secondary to hypothermia, terminal neoplastic disease, inmates and absence of informed consent.

### Study protocol

CA was managed in all patients according to European Resuscitation Council Guidelines 2015. Resuscitation was initiated on-site by emergency medical team members [20, 21]. All the study patients were treated with conventional cardiopulmonary resuscitation (CPR). Extracorporeal cardiopulmonary resuscitation (ECPR) has not been applied to any patient in our study. Fluid infusion and vasopressors/inotropes, alone or in combination (based on individual clinician judgment) were administered to maintain mean arterial pressure ≥ 65 mmHg and urine output of ≥ 0.5 ml/kg/hour. After successful resuscitation, for comatose patients that were able to maintain a systolic blood pressure above 90 mmHg (mean arterial pressure—MAP ≥ 65 mmHg, with or without vasopressors) and did not present with sepsis, controlled therapeutic hypothermia was administered in the first 24 hours. A core temperature between 34–35°C was achieved using ice bags and cooling blankets. Reheating was slow at a rate of 0.25–0.5C°/hour. Hyperthermia, convulsions and hyperglycemia were avoided and treated according to current resuscitation protocols [3, 22].

Post-CA shock was defined as need for continuous vasopressors/inotropes infusion to maintain MAP > 65 mmHg despite adequate fluid loading, for more than 6 hours after obtaining ROSC. The cause of death was defined as related to post-CA shock (death occurred as a direct consequence of shock, including subsequent multiorgan failure), to hypoxic-ischemic brain injury or to sepsis.

Clinical data such as initial Sequential Organ Failure Assessment (SOFA) score on admission to ED [23], highest SOFA (highest score registered every 24 hours after admission for the first 3 days or until death), age, gender, primary rhythm of CA, duration of resuscitation, the presence or absence of cardiovascular and non-cardiovascular comorbidities were registered.

### Blood samples and laboratory assays

Blood samples were drawn from a separate peripheral vein at admission in ED (0-time interval), and at 6, 12, 24, 48- and 72-hours following resuscitation. To collect blood samples, 5 ml serum separator clot activator biochemistry vacutainers were used. The hemolyzed samples were excluded and blood samples were immediately repeated. Samples were centrifuged at 3000 rotation/minutes during the first 60 minutes after prelevation and were stored at -70°C. Subsequently, serum concentrations of biomarkers resistin, S-100B and NSE were determined using a quantitative sandwich immunoassay technique (ELISA; BioVendor, LM, Czech Republic) according to the manufacturer’s instructions.

### Statistical analysis

Statistical analyzes were performed using the MedCalc Statistical Software version 18.2.1 (MedCalc Software bvba, Ostend, Belgium; http://www.medcalc.org/; 2018). Quantitative data were expressed as median and interquartile range (non-normal distribution), and qualitative data were characterized by frequency and percentage. Comparisons between groups were performed using the Chi-Square test, Fisher’s exact test, or Mann-Whitney test, whenever appropriate. To identify predictors of death, baseline characteristics were presented stratified by 30-day survival. For each marker we calculated the area under the curve using the trapezoidal method. This included all determinations of serum concentrations of biomarkers studied over time from the first measurement up to 12, 24, 48 or 72 hours. In order to find out how accurate a marker differentiates the deceased form the survivors, we used the area under the receiver operating characteristics (AUROC) curves. The cut-off value for each biomarker was calculated where specificity and sensibility were maximal. A predictive score for the mortality at 30 days was created with a logistic regression and it included the best performing markers. The score was calculated using the following formula: score = exp (c + b_i_x_i_) / 1+ exp(c + b_i_x_i_); where exp is the base of natural logarithms; c is the constant of the logistic regression; b is the coefficient of the predictor variable; x is value of the marker. A p value < 0.05 was considered statistically significant.

## Results

**Fig 1:**
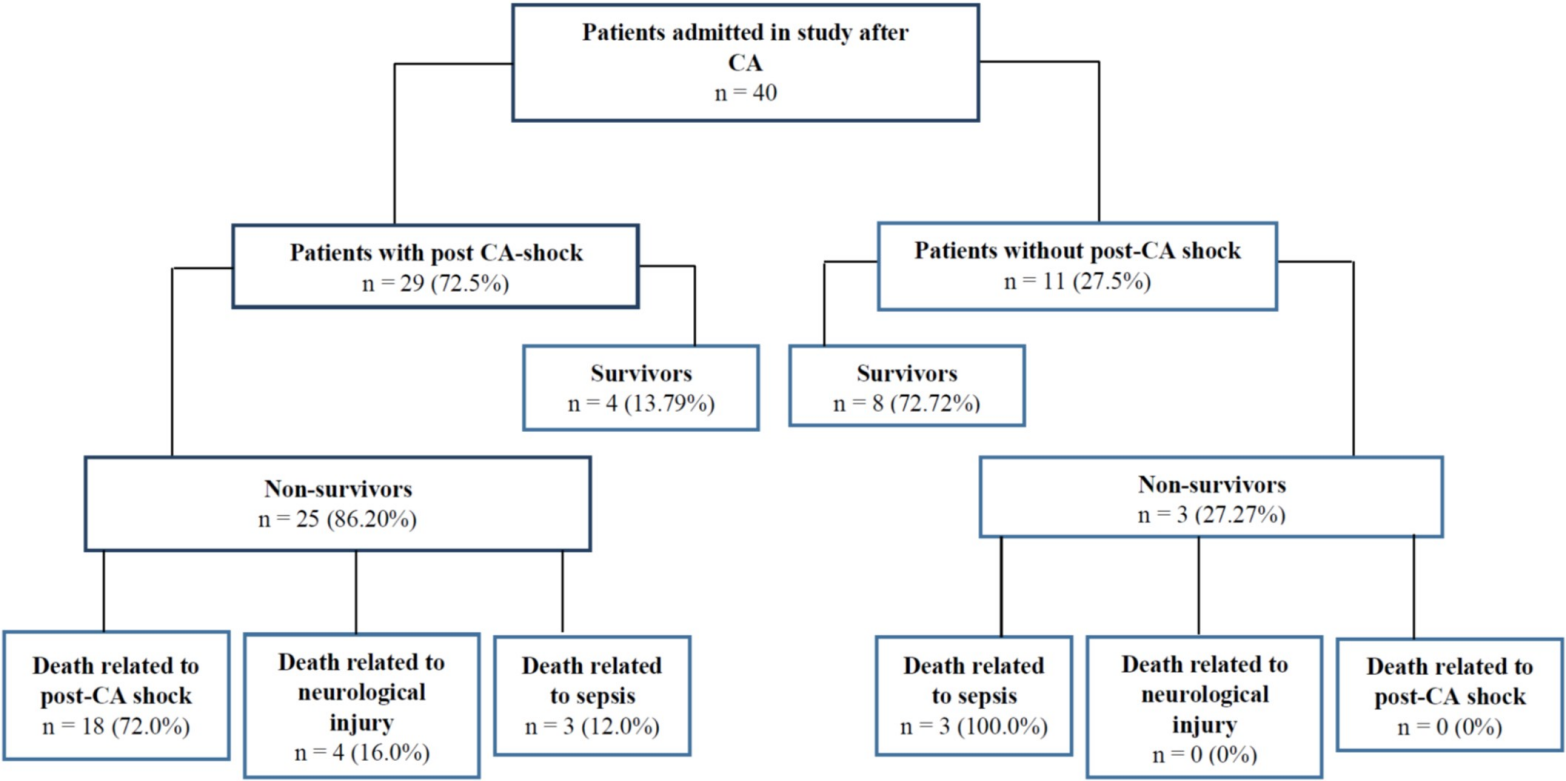
Flowchart.

Of the 152 consecutive patients resuscitated after CA and admitted to the ED, 40 were eligible and have been included in the study with a median age of 67 years (IQR: 59.2 to 76). In this study group 28 (70.0%) patients were men. Most frequent rhythms of CA were asystole and pulseless electrical activity, found in 28 cases (70.0%), with a median duration of resuscitation of 15 minutes (IQR: 7.75 to 28.75). Most patients developed post CA-shock after initial resuscitation [n = 29 (72.5%)] with a high mortality rate [n = 25 (86.2%)], especially due to post-CA shock [n = 18 (72.0%)] (Fig 1).

Cardiovascular disease was the main cause of CA with 29 (72.5%) cases and acute myocardial infarction (40.0%) was the most common presentation (all patients receiving coronary revascularization immediately after resuscitation) (Table 1).

**Table 1:** Causes of CA registered in study patients and the causes of death at 30 days in relation to post-CA shock, neurological injury and sepsis.

Causes of cardiac arrest n = 40 (100%)	Non-survivors n = 28 (70.0%)		
	Post-CA shock	Neurological	injury Sepsis
**Acute myocardial infarction** **16 (40.0%)**	4 (14.80%)	3 (10.71%)	0 (0%)
**Pulmonary Embolism** **2 (5.0%)**	2 (7.14%)	0 (0%)	0 (0%)
**Acute pulmonary edema** **4 (10.0%)**	4 (14.28%)	0 (0%)	0 (0%)
**Rhythm disorders related to heart disease** **7 (17.5%)**	1 (3.57%)	0 (0%)	3 (10.71%)
**Rhythm disorders related to electrolyte imbalances** **2 (5.0%)**	2 (7.14%)	0 (0%)	0 (0%)
**Acute respiratory failure** **3 (7.5%)**	1 (3.57%)	0 (0%)	2 (7.14%)
**Stroke** **2 (5.0%)**	0 (0%)	1 (3.57%)	1 (3.57%)
**Septic shock** **4 (10.0%)**	4 (14.8%)	0 (0%)	0 (0%)

Of the 40 enrolled patients, 12 (30.0%) survived and 28 (70.0%) died before 30 days: 6 patients (15.0%) died in the first 24 hours after admission but not within the first 6 hours, while other 6 patients (15.0%) died before reaching 48 hours of admission; 3 patients (7.5%) died between 48–72 hours and 13 patients (32.5%) died after 72 hours of admission but before completing 30 days of follow-up.

Cardiovascular comorbidities and higher disease severity scores were registered in non-survivors as otherwise expected. In survivors, the initial rhythm at presentation was ventricular fibrillation/pulseless ventricular tachycardia, with a shorter duration of resuscitation compared to those who died most often presented with asystole/pulseless electrical activity (Table 2).

**Table 2:** Baseline characteristics and SOFA scores stratified by 30 days survival.

Characteristics		AUC	Non-survivors (n = 28)	Survivors (n = 12)	p
**Age, years, median (IQR)**		0.735	69.0 (IQR:66.0 to76.7)	60.50 (IQR:45.0 to 66.7)	0.02
**Sex, n (%)**	Female	–	9 (32.1)	3 (25.0)	0.72
	Male	–	19 (67.9)	9 (75.0)	
**Primary rhythm (%)**	VF/VT	–	2 (7.1)	10 (83.3)	<0.001
	Asystole/PEA	–	26 (92.9)	2 (16.7)	
**Duration of CPR, minutes, median (IQR)**		0.713	15.0 (IQR:10.0 to 30.0)	6.0 (IQR:4.2 to 20.0)	0.03
**Cardiovascular comorbidities, n (%)**		–	22 (78.6)	4 (33.3)	0.01
**Non -cardiovascular comorbidities, n (%)**		–	14 (50.0)	4 (33.3)	0.49
**SOFA score at admission, median (IQR)**		0.815	15.0 (IQR:13.0 to 17.0)	11.0 (IQR:6.2 to 13.0)	0.002
**TH, n (%)**		–	22 (81)	5 (18.5)	0.03
**Lactate at admission, median (IQR)**		0.890	11.3 (IQR: 10.1 to 13.3)	5.7 (IQR: 2.8 to 9.1)	<0.001
**Creatinine at admission, median (IQR)**		0.734	1.4 (IQR: 1.1 to 1.9)	1 (IQR: 0.9 to 1.3)	0.008

PEA = pulseless electrical activity; VF = ventricular fibrillation, VT = ventricular tachycardia

CPR = cardiopulmonary resuscitation; IQR = interquartile range

SOFA = Sequential Organ Failure Assessment Score; Highest SOFA = the highest SOFA score recorded in the first 72 hours

TH = therapeutic hypothermia

### Biomarkers assay

At each time interval, median serum levels of resistin and S-100B were significantly higher in non-survivors compared to survivors (p < 0.05). For NSE, plasma levels were significantly lower in survivors compared to non-survivors at 48 and 72 hours, respectively (p < 0.05) (Table 3).

**Table 3:** Median serum levels and AUC of resistin, S-100B and NSE at admission and during the first 72 hours.

Variable	Time interval	AUC	Non-survivors (n = 28)	Survivors (n = 12)	p
**Resistin (ng/ml)**	0 hours	0.751	9.35 (IQR: 5.6 to 13.7)	5.80 (IQR: 1.9 to 6.9)	0.01
	6 hours	0.807	11.80 (IQR: 7.1 to 19.8)	4.30 (IQR: 3.2 to 9.3)	0.002
	12 hours	0.875	15.85 (IQR: 10.8 to 24.2)	4.50 (IQR: 1.6 to 11.2)	< 0.001
	24 hours	0.795	16.50 (IQR: 10.2 to 26.5)	6.75 (IQR: 3.6 to 9.6)	0.001
	48 hours	0.779	13.45 (IQR: 5.2 to 21.0)	3.65 (IQR: 1.6 to 7.4)	0.004
	72 hours	0.763	11.00 (IQR: 6.2 to 17.8)	5.35 (IQR: 2.5 to 8.0)	0.02
**S-100B (pg/ml)**	0 hours	0.798	52.80 (IQR: 15.7 to 122.8)	5.95 (IQR: 3.7 to 20.9)	0.003
	6 hours	0.918	40.90 (IQR: 4.8 to 190.3)	4.80 (IQR: 3.3 to 8.9)	< 0.001
	12 hours	0.839	79.90 (IQR: 9.5 to 165.2)	4.60 (IQR: 3.3 to 12.8)	0.001
	24 hours	0.782	56.50 (IQR: 10.9 to 199.5)	4.60 (IQR: 3.3 to 12.8)	0.001
	48 hours	0.776	31.50 (IQR: 11.6 to 201.5)	6.60 (IQR: 3.6 to 15.7)	0.009
	72 hours	0.798	25.10 (IQR: 7.8 to 80.7)	4.80 (IQR: 3.3 to 12.8)	0.01
**NSE (ng/ml)**	0 hours	0.509	7.30 (IQR: 3.27 to 15.87)	8.05 (IQR: 2.10 to 18.15)	0.92
	6 hours	0.618	15.40 (IQR: 4.77 to 47.77)	10.85 (IQR: 3.92 to 19.62)	0.24
	12 hours	0.652	20.05 (IQR: 6.52 to 80.77)	12.10 (IQR: 1.77 to 20.12)	0.13
	24 hours	0.506	19.85 (IQR: 6.65 to 97.65)	10.60 (IQR: 4.67 to 20.17)	0.17
	48 hours	0.779	72.20 (IQR: 9.70 to 211.87)	5.40 (IQR: 1.35 to 11.70)	0.006
	72 hours	0.750	67.00 (IQR: 4.75 to 194.60)	4.00 (IQR: 1.90 to 23.95)	0.03

AUC = area under the curve; IQR = interquartile range

Comparing serum concentrations of biomarkers at different time intervals, in non-survivors and survivors, shown that levels were significantly higher in non-survivors for resistin and S-100B (p < 0.05), with a relatively constant AUCs for resistin determinations (Table 4).

**Table 4:** Comparison of area for markers measurements between non-survivors and survivors.

Variable		AUC	Non-survivors (n = 28)	Survivors (n = 12)	P
**Area for resistin ng x h/ml**	0–12 hours (3 measurements)	0.792	26.0 (IQR: 14.0–37.5)	9.5 (IQR: 4.2–22.5)	<0.001
	0–24 hours (4 measurements)	0.788	27.2 (IQR: 18.3–37.5)	14.9 (IQR: 8.3–21.8)	0.001
	0–48 hours (5 measurements)	0.795	22.0 (IQR: 14.5–38.7)	12.7 (IQR: 5.3–15.9)	0.003
	0–72 hours (6 measurements)	0.814	44.9 (IQR: 30.5–71.9)	23.4 (IQR: 15.4–33.7)	0.008
**Area for S-100B pg x h/m**l	0–12 hours (3 measurements)	0.833	75.0 (IQR: 24.5–233.0)	12.0 (IQR: 7.2–30.5)	<0.001
	0–24 hours (4 measurements)	0.647	64.8 (IQR: 15.5–129.3)	23.2 (IQR: 12.9–30.2)	0.011
	0–48 hours (5 measurements)	0.692	55.1 (IQR: 22.6–251.8)	26.4 (IQR: 15.1–31.1)	0.041
	0–72 hours (6 measurements)	0.750	122.5 (IQR: 38.0–372.0)	37.8 (IQR: 22.5–73.8)	0.034
**Area for NSE** **ng x h/ml**	0–12 hours (3 measurements)	0.455	19.0 (IQR: 9.0–61.5)	27.0 (IQR: 6.0–38.0)	0.215
	0–24 hours (4 measurements)	0.551	39.8 (IQR: 11.2–47.5)	23.5 (IQR: 10.7–52.2)	0.097
	0–48 hours (5 measurements)	0.763	123.0 (IQR: 28.5–313.5)	19.0 (IQR: 6.25–37.5)	0.008
	0–72 hours (6 measurements)	0.737	108.0 (IQR: 25.0–318.0)	16.5 (IQR: 8.5–48.2)	0.044

For predicting the mortality at 30 days, we created several predictive models using logistic regressions (Table 5). In each model we included 2 or 3 variables that achieved the highest AUC alone (Tables 3 and 4) in order to established which score could better predict the mortality. The score was calculated using the equation described in materials and methods and the coefficients were provided by the regression.

**Table 5:** Logistic regression for 30-days mortality.

Variables		B	P	OR	95% C.I. for OR	
					Lower	Upper
**Model for resistin and S-100B combined score at 12 hours**	**S-100B pg/ml at 12 hours**	0.058	0.1	1.060	.975	1.153
	**Resistin ng/ml at 12 hours**	0.262	0.020	1.300	1.042	1.621
	**Constant**	-3.057	0.01	0.047	–	–
**Model for resistin, NSE and S-100B combined score at 12 hours**	**S-100B pg/ml at 12 hours**	0.136	0.2	1.146	0.899	1.461
	**Resistin ng/ml at 12 hours**	0.374	0.02	1.453	1.050	2.010
	**NSE ng/ml at 12 hours**	-0.024	0.1	0.977	0.949	1.006
	**Constant**	-4.078	0.04	0.017	–	–
**Model for area for resistin and S-100B combined score between 0–12 hours**	**Area for resistin 0–12 hours**	0.135	0.02	1.145	1.019	1.285
	**Area for S-100B 0–12 hours**	0.033	0.1	1.034	0.993	1.076
	**Constant**	-3.423	0.01	0.033	–	–
**Model for areas for resistin, NSE and S-100B combined score between 0–12 hours**	**Area for resistin 0–12 hours**	0.204	0.02	1.226	1.026	1.465
	**Area for S-100B 0–12 hours**	0.055	0.1	1.056	0.983	1.134
	**Area for NSE 0–12 hours**	-0.014	0.1	0.986	0.969	1.003
	**Constant**	-4.530	0.03	0.011	–	–
**Model for resistin and S-100B at 6 hours and lactate at 0 hours combined score**	**Lactate mmol/L at 0 hours**	1.880	0.2	6.553	0.206	208.9
	**S-100B pg/ml at 6 hours**	1.415	0.3	4.116	0.261	64.824
	**Resistin ng/ml at 6 hours**	1.269	0.2	3.559	0.493	25.701
	**Constant**	-37.919	0.2	0	–	–

After calculating the scores, we used the AUROC to determine which biomarker could differentiate more accurately the non-survivors from the survivors. The most accurate scores are presented in Table 6. We did not find statistically significant differences between any of the AUCs of the scores.

**Table 6:** AUCs for the predictive scores.

Predictive score	AUC (CI95%)	Cut-off	Sensitivity (CI 95%)	Specificity (CI 95%)	p
**Resistin and S100B combined score at 12 hours**	0.938 (0.813–0.989)	> 0.65	85.71 (67.3–96.0)	91.67 (61.5–99.8)	<0.001
**Resistin, NSE and S100B combined score at 12 hours**	0.955 (0.839–0.995)	> 0.73	82.14 (63.1–93.9)	100.00 (73.5–100.0)	<0.001
**Area for resistin and S100B combined score between 0–12 hours**	0.937 (0.813–0.989)	> 0.58	82.14 (63.1–93.9)	91.67 (61.5–99.8)	<0.001
**Area for resistin, NSE and S100B combined score between 0–12 hours**	0.958 (0.843–0.996)	> 0.69	85.71 (67.3–96.0)	100.00 (73.5–100.0)	<0.001
**Resistin and S100B at 6 hours and lactate at 0 hours combined score**	0.994 (0.901–1)	> 0.57	96.4 (81.7–99.9)	100 (73.5–100.0)	<0.001

When we compared the predictive value of resistin alone at 12 hours with the combination of S-100B and resistin at 12 hours we found a p = 0.07. The sensitivity was almost the same (85.7%) but the specificity was lower for resistin (75% vs. 91.6%). When we compared the predictive value of resistin alone at 12 hours with the area for resistin and S-100B combined score between 0–12 hours we found a p = 0.08. When we compared the predictive value of resistin alone at 12 hours with the resistin, NSE and S-100B combined score at 12 hours we found a p = 0.1. When we compared the predictive value of resistin alone at 12 hours with area for resistin, NSE and S-100B combined score between 0–12 hours we found a p = 0.1. When we compared the predictive value of resistin alone at 12 hours with resistin and S-100B at 6 hours and lactate at 0 hours combined score we found a p = 0.03.

We examined the usefulness of the markers for predicting the mortality at 24 hours, since guidelines usually cannot indicate the predictors for such an early event. Only high resistin values had a statistically significant association with the 24-hours morality: at 0 hours (p = 0.008), at 6 hours (p = 0.02) and at 12 hours (p = 0.03).

**Fig 2:**
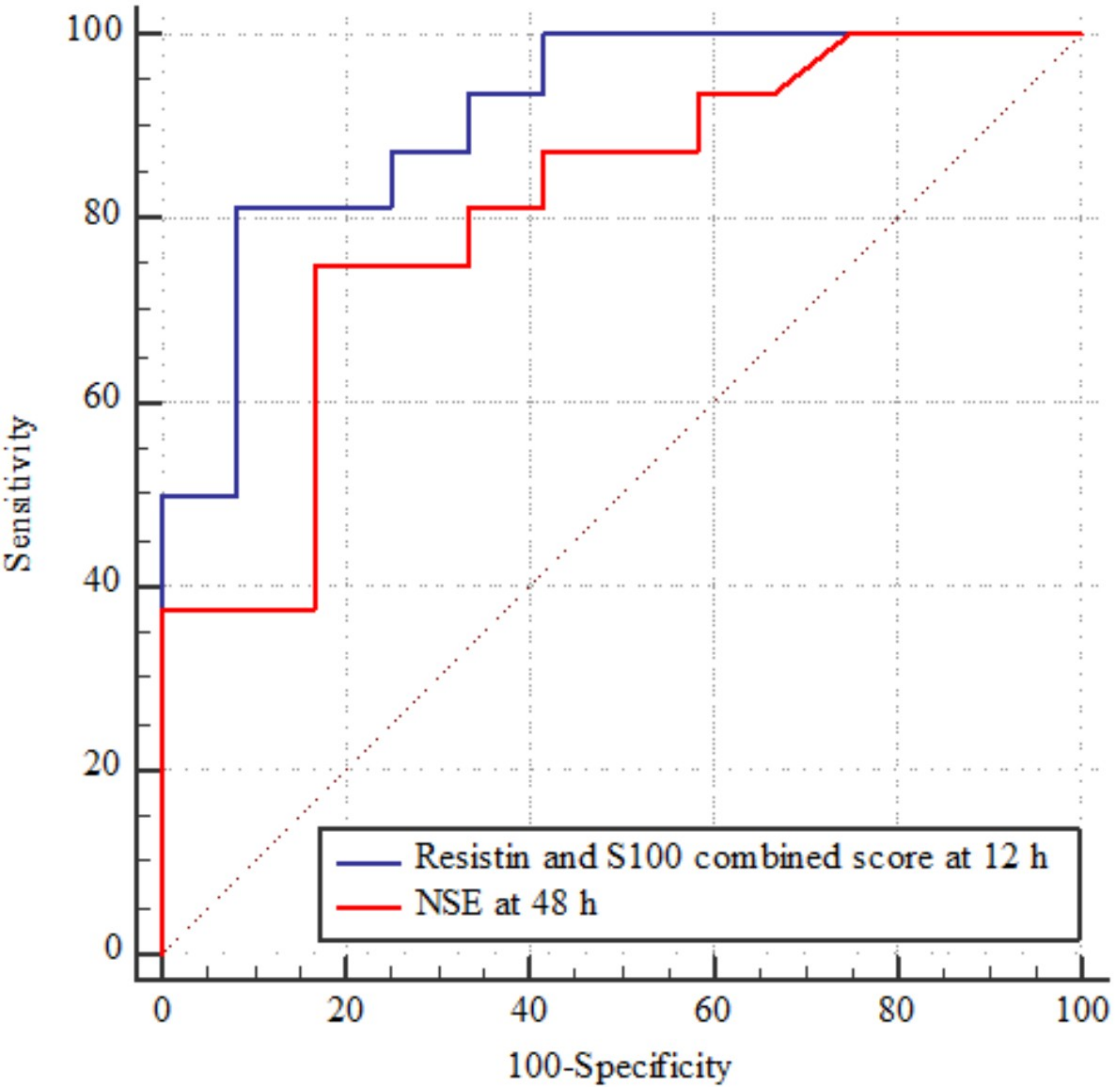
Comparison between AUCs for differentiating the 30-days mortality.

Considering that at this point the NSE is useful for predicting mortality, if is measured in dynamics and after 48–72 hours, we compared it with the score that combined resistin and S100 at 12 hours (Fig 2). We obtained an AUC of 0.911 (0.742 to 0.985) for the score and an AUC of 0.810 (0.618 to 0.932) for NSE alone. The difference between AUCs was not statistically significant (p = 0.2).

## Discussions

In the last few years it has been shown that resistin levels are well correlated with prognosis of critically ill patients [18]. This correlation may be attributed to the fact that resistin is involved in the release of increased levels of pro-inflammatory biomarkers—IL-6, IL-12 and TNF-ɑ- that are involved in organ failure caused by sepsis [14, 16].

Taking into consideration that an inflammatory process is involved in hypoxic-ischemic organ injury after CA, we investigated a possible correlation between resistin serum levels and the outcome in resuscitated patients and possible predictive value of resistin combined with S-100B and NSE. To our knowledge this is the first study to evaluate resistin as a biomarker for the outcome of resuscitated patients.

Previous studies have focused on the role of NSE and S-100B protein [3, 22] in predicting outcome in patients resuscitated after CA, in order to direct maximal resources for the treatment of these patients [4–6, 9–12], although the time interval for markers assay seems to be crucial in defining their role for predicting patients’ outcome [3, 5]. Today these markers are included in resuscitation guidelines.

Stammet P et al., found that NSE levels were well correlated with the outcome in resuscitated patients after CA, as an indicator of increased risk of death and poor neurologic outcome [5]. Similarly, Wiberg S et al. showed that NSE is a strong marker for predicting mortality at 48 hours after resuscitation, in patients with initially successfully resuscitated CA although a cut-off value with 100% specificity (false positive rate of 0) and high sensitivity could not yet be determined [4, 6].

Our study found similar results, showing that NSE levels were significantly elevated in non-survivors, with the highest sensitivity and specificity for predicting mortality if measured at 48 hours from the event.

Wiberg S. et al showed that S-100B is an important marker in predicting mortality after successfully resuscitated CA [6], although it’s role in resuscitation is not fully attributable. Similar results are reported by us in this study. We showed that S100B levels were significantly higher in patients who died, at all-time intervals, making S100B a highly predictive and more accurate biomarker for 30-day mortality than NSE.

For resistin, our results showed that levels (including median serum concentrations levels) were significantly increased at each time interval of assay in non-survivors compared to survivors with relatively constant AUCs and high predictive value for 30-day mortality compared with NSE.

Starting from these results, for predicting the mortality at 30 days, we created several models following the pattern that could better differentiate the non-survivors from the survivors. Taking into account the cost-effective ratio (the number of determinations over time, the current cost of biomarker kits), we could demonstrate that six biomarkers determinations that combined resistin with S100 in the first 12 hours is sufficient for early prediction of outcome in patients who survived after a CA (deceased versus survivor).

We must agree and point out, that both biomarkers had a lower sensitivity than specificity for all determinations (although they are very close). This reinforces the suggestion that interpreting the values of these biomarkers in relation to post-cardiac arrest survival should be part of a multifactorial approach of the clinician.

One of the biological variables of interest in determining the prognosis of critically ill patients is serum lactate. Recent studies demonstrate that elevated serum lactate levels determined upon patient arrival in the emergency department is associated with unfavorable prognosis of patients who presented an episode of CA [24, 25]. The results were promising and from the many combinations of biomarkers lactate value at arrival and serum resistin and S100B values at 6 hours best predict mortality (AUC = 0.994, Sp = 100%, Se = 96.4%, p <0.001). Although we are tempted to believe that we have found the ideal predictive combination, the fairly wide confidence interval of the regression variables on which we built the score impose the need for a larger group of patients to increase the accuracy of the model.

Regarding the usefulness of biomarkers in the prediction of 24-hour early mortality in a patient who initially survived a successfully resuscitated CA, resistin was the only one that was statistically significantly associated with morality.

All of these findings support the idea that resistin may be an important future biomarker in predicting early prognosis after resuscitation, due to its role in the inflammatory processes underlying the hypoxic-ischemic organ injuries after a CA. By combining resistin with S-100B and with S-100B and lactate, predictive value of these biomarkers increased.

Our study is limited by the inclusion of a small number of resuscitated patients, comparable however with other similar studies. At the same time, we could not validate the predictive score due to the difficulty in recruiting patients. We mention that all the study patients were treated with conventional CPR and the results of the study could not extrapolate to patients treated with ECPR.

## Conclusion

In our study, serum levels of resistin alone or a combination of resistin with S-100B or resistin with S-100B and lactate were highly predictive of 30 days mortality in resuscitated patients after CA. Further studies on larger number of patients are needed to confirm our hypothesis.

## Supporting information

S1 Database (XLSX)
